# Genetic, physiological and biochemical characterization of *Bacillus* sp. strain RMB7 exhibiting plant growth promoting and broad spectrum antifungal activities

**DOI:** 10.1186/s12934-014-0144-x

**Published:** 2014-10-24

**Authors:** Saira Ali, Sohail Hameed, Asma Imran, Mazhar Iqbal, George Lazarovits

**Affiliations:** National Institute for Biotechnology and Genetic Engineering (NIBGE), P.O. Box 577, Jhang Road, Faisalabad, 38000 Pakistan; A&L Biologicals, Agroecology Research Service Centre, 2136 Jet stream Road, London, ON N5V 3P5 Canada; Department of Biology, University of Western Ontario, London, ON Canada

**Keywords:** Biopesticides, Bacillus, Biofertilizers, Iturin A, Surfactin, Mass chromatography

## Abstract

**Background:**

Plant growth promoting rhizobacteria (PGPR) are functionally diverse group of bacteria having immense potential as biofertilizers and biopesticides. Depending upon their function, they may serve as partial replacements for chemical fertilizer or pesticides as an eco-friendly and cost-effective alternatives as compared to their synthetic counterparts. Therefore, isolation, characterization and practical evaluation of PGPRs having the aforementioned multifaceted beneficial characteristics, are essentially required. This study describes the detailed polyphasic characterization of *Bacillus* sp. strain RMB7 having profound broad spectrum antifungal activity and plant growth promoting potential.

**Results:**

Based on 16S rRNA gene sequencing, strain RMB7 was identified as *Bacillus* specie. This strain exhibited the production of 8 mg. L^−1^of indole-3-acetic acid (IAA) in tryptophan-supplemented medium. It was able to solubilize 50.6 mg. L^−1^ tri-calcium phosphate, reduced 601ηmol acetylene h^−1^/vial and inhibited >70% growth of nine fungal phytopathogens tested *in vitro*. Under natural pathogen pressure, inoculation with strain RMB7 and RMB7-supernatant conferred resistance by arugula plant against *Pythium irregulare* with a concurrent growth improvement over non-inoculated plants. The T-RFLP analysis based on 16S *rRNA* gene showed that inoculation with RMB7 or its supernatant have a major impact on the indigenous rhizosphere bacterial population. Mass spectrometric analysis revealed the production of lipopeptide surfactins as well as iturin A presence in crude extract of RMB7. PCR-amplification further confirmed the presence of genes involved in the biosynthesis of these two bioactive lipopeptide compounds.

**Conclusions:**

The data show that *Bacillus* sp. strain RMB7 has multifaceted beneficial characteristics. It may be an ideal plant growth promoting as well as biocontrol agent, for its integrated use in disease and nutrient management strategies.

## Background

Due to food safety issues and increasing environmental concerns, the usage of biofertilizers and biopesticides is gaining attention in the agriculture sector world-wide [1]. The antagonistic or plant growth promoting rhizobacteria (PGPR) and their bioactive antimicrobial compounds are considered as environmental friendly and easily biodegradable. Hence, they can be the key players in the sustainable agriculture [2]. PGPR enrich the soil environment with micro- and macro-nutrients by means of nitrogen fixation, phosphate/potassium/zinc solubilisations or mineralization, release of plant growth regulating substances, degradation of organic matter [3] and can control plant pathogens, especially the fungus, by producing antifungal metabolites or through induction of systemic resistance [4-7]. PGPR stimulate plant growth and productivity by improving nutrient supply, contributing in plant defense and protection through triggering various growth- and defense-related genes to induce cellular response, consequently improving the crop yield [2].

Currently, PGPR inoculants consist of the bacteria mainly from the genus *Rhizobium* (symbiotic N_2_-fixing bacteria), *Azospirillum* (free living N_2_-fixing bacteria), *Bacillus* (phosphate-solubilizing bacteria and biocontrol agents), *Pseudomonas* and *Trichoderma* (biocontrol agents) [8-10]. *Bacillus subtilis* is a common soil and rhizosphere resident bacteria having dual beneficial properties of plant growth promotion as well as biocontrol activities [11,12]. Almost 5% of its genome is devoted for the production of antibiotics and many strains from this genus have shown the potential to produce about two dozen structurally diverse antimicrobial compounds [11-13]. Most active and useful among these compounds are the cyclic lipopeptides of three families: surfactin, iturin and fengycin. These lipopeptides not only show antifungal and biocontrol activity but play vital role in root colonization of *Bacillus* [1].

Surfactin family consists of about 20 different lipopeptides [14], which are heptapeptides interlinked with *β*-hydroxyl fatty acid to from a cyclic lactone ring structure [15]. The amino acids are arranged in the chiral sequence LLDLLDL with hydrophobic amino acids mostly located at positions 2, 3, 4, 6, and 7, while hydrophilic amino acids are generally located at sequence positions 1 and 5 as glutamyl and aspartyl, respectively [16]. Due to their amphiphilic nature, surfactins can readily associate and tightly anchor into lipid layers, and promote colonization [14]. Recent studies have shown that surfactin could induce plant systemic resistance [17]. Three genes *srfA, sfp,* and *comA* are found to be involved in the synthesis of surfactin of which *sfp* gene is being used as marker for identification of surfactin producing *Bacillus* strains [18].

Iturins are one of the most popular antifungal lipopeptides used for the biocontrol of fungal plant diseases [19]. Antifungal activity of iturins results in the formation of pores in the cell membrane which cause leakage of potassium ions. They interact with sterols and have been shown to form aggregates in the phospholipid membrane [20]. Iturin A, C, bacillomycin D, F, L, LC and mycosubtilin are seven main variants within the iturin family. They are heptapeptides linked to a β-amino fatty acid chain with a length of 14 to 17 carbons. Iturins are also biosurfactants but their biological activity differs from surfactins: they display strong *in vitro* and *in vivo* antifungal activities [21,22]. This fungitoxicity relies on their membrane permeability. Iturin derivatives enhance the invasive growth of the producing strain, and thus, by these two mechanisms participate in plant protection against phytopathogens [23]. Iturin A operon is more than 38 kb long and consists of four open reading frames, *itu D, itu A, itu B* and *itu C*. The *itu D* gene encodes a putative malonyl coenzyme A transacylase, which plays role in biocontrol activity [24].

*Bacillus* spp*.* have been pursued for the specific production of various chemical entities such as polyhydroxyalkanoates [25], isobutanol [26], antibacterial polyketides macrolactin, bacillaene and difficidin [27], cyclic lipopeptides surfactin, iturin A and fengycin as well as the iron-siderophore bacillibactin [27,28]. It has also been extensively investigated as PGPR agent for both; biofertilization applications (N_2_ fixation, siderophore production, P solubilization, IAA production) and/or biocontrol applications (antibiosis, secretion of lytic enzymes, and induction of systemic resistance in host plant) [29].

Previously, studies have been mainly focused on the isolation and characterization of microbes having either PGPR or biocontrol activities. However, a very limited data is available, where most of the aforementioned beneficial aspects of bacterium have been fully explored in a single study [29,30]. The strains having either PGPR or biocontrol ability are usually applied in mix consortium to get synergistic effect in field [29]. The use of single strain inoculum, if available, however, may be more user friendly as compared to multi-strain consortium. Among PGPR, *Pseudomonas aeruginosa* and *Pseudomonas aurantiaca* strains exhibiting broad spectrum antifungal activity and biofertilizer traits have been described along-with detailed characterization of their antifungal metabolites produced and identification of the putative genes involved in the biocontrol mechanism [31,32]. Endophytic *B. subtilis* strain ALB629 having growth promoting effects on cacao as well as antagonistic effect against the phytopathogenic fungi *Moniliophthora perniciosa* and *Colletotrichum* spp. has been described but the antimicrobial compounds involved were not identified [33]. Multifaceted *Bacillus* isolates RM-2 and BPR7 having PGPR and antagonistic activities have also been described but the antifungal metabolites, the mechanism of biocontrol and *in vivo* biocontrol potential was not documented [34,35].

With the aim to identify bacteria having multifaceted beneficial applications for crops improvement, we isolated 127 bacteria from rhizosphere of maize growing fields from geographically underexplored foot hills of Himalaya region (Azad Jammu & Kashmir, Pakistan). These bacteria were screened for their PGPR activities. Among them, the bacterium named RMB7 having the best PGPR and biocontrol activities, was subjected to details characterization. This bacterium exhibited the significant broad-spectrum antifungal activities (both in plate assay as well as in planta studies). The antifungal metabolites produced by bacterial strain RMB7 were comprehensively characterized using a polyphasic approach. The biocontrol activity of RMB7 was tested on Arugula plant against *Pythium irregulare* which is a broad-spectrum pathogen infecting wide-range of crops. The ultimate objective was to select the most efficient antagonistic PGPR having multifaceted benefits that can be used as biofertilizer as well as biopesticide for various crops cultivated in South Asian region.

## Results

### Isolation and identification of *Bacillus* sp. strain RMB7

The rhizobacterial isolate RMB7, obtained from the roots of maize plant growing in the foot hills of Himalaya, was characterized as *Bacillus* sp. on the basis of 16S rRNA gene sequencing. The obtained 1260 bp fragment of RMB7 showed 97% homology to 16S rRNA gene of *B. subtilis* subsp. inaquosrum strain BGSC 3A28 (GenBank accession # NR-104873.1), *B. Subtilis* strain BCRC 10255 (GenBank accession # NR-116017.1) and *B. subtilis* subsp. subtilis 6051-HGW complete genome (GenBank accession # CP003329.1). The gene sequence of RMB7 was submitted to GenBank data base and accession number was assigned (JQ425166).

### Physico-chemical and functional characterization of *Bacillus* sp. strain RMB7

Cells of bacterial strain RMB7 appeared Gram positive, rod-shaped, motile and single cells under light microscope. On Luria-Bertani (LB) agar, RMB7 formed circular, flat, smooth, opaque, off-white colonies with undulate margins. RMB7 solubilized 50.6 ± 3.4 mg. L^−1^ tri-calcium phosphate within seven days, produced 8 ± 1.0 ppm IAA in the presence of tryptophan, reduced 601.5 ηmol h.^−1^ vial^−1^ of acetylene to ethylene on nitrogen free malate medium (NFM), utilized 1-aminocyclopropane-1-carboxylic acid (ACC) as carbon source and formed biofilm on the glass surface.

### Antifungal and phenotype assays

**Figure 1:**
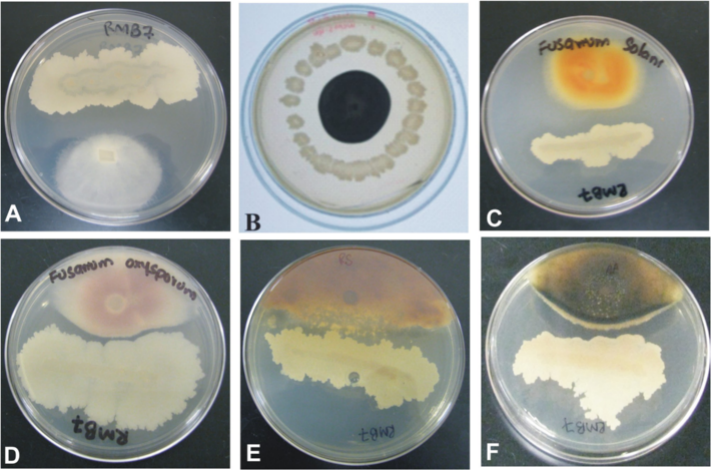
**Antifungal dual plate assay of** ***Bacillus*** **sp. strain RMB7 with: (A)** ***Pythium irregulare*** **(B)** ***Aspergillus niger*** **(C)** ***Fusarium solani*** **(D)** ***Fusarium oxysporum*** **(E)** ***Rhizoctonia solani*** **and (F)** ***Alternaria alternata.***

Strain RMB7 exhibited broad-spectrum antifungal activity *in vitro* (Figure 1) showing >70% inhibition of mycelia growth of all the fungal pathogens tested. Growth inhibition was variable for each fungus *e.g*., *Aspergillus niger* (76%)*, Aspergillus flavus* (75%)*, Colletotrichum gloeosporioides* (78%)*, Colletotrichum falcatum* (77%)*, Fusarium oxysporum* (71%)*, Fusarium moniliforme* (79%)*, Rhizoctonia solani* (70%)*, Pythium ultimatum* (83%) and *Pythium irregulare* (85%). Strain RMB7 was able to digest skim milk, colloidal chitin and cellulose, indicating protease, chitinase and cellulase activities. It showed the production of diffusible antibiotics and siderophores but did not show the production of volatile antibiotics or HCN.

### *In vivo* plant-inoculation assay

**Figure 2:**
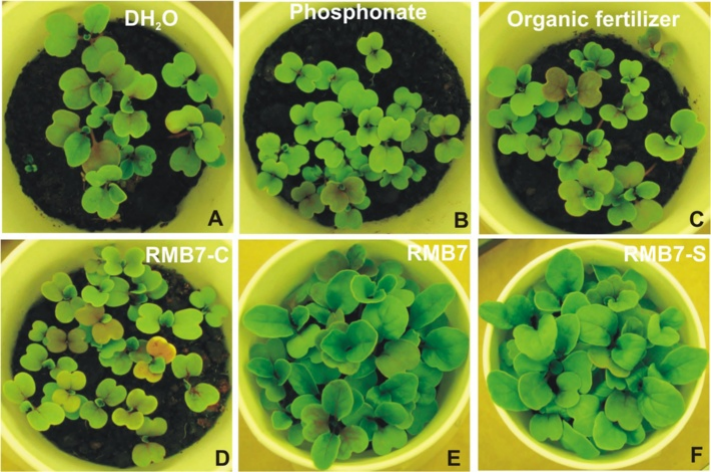
**Pot view of Arugula grown in** ***P. irregulare*** **infested soil provided with different treatments: (A) Autoclaved distilled water (DH**_**2**_ **O); (B) Fungicide (P); (C) Organic fertilizer (OF); (D) RMB7 cells (C); (E) RMB7 cells + supernatant (CS); (F) RMB7 supernatant (S).**

**Figure 3:**
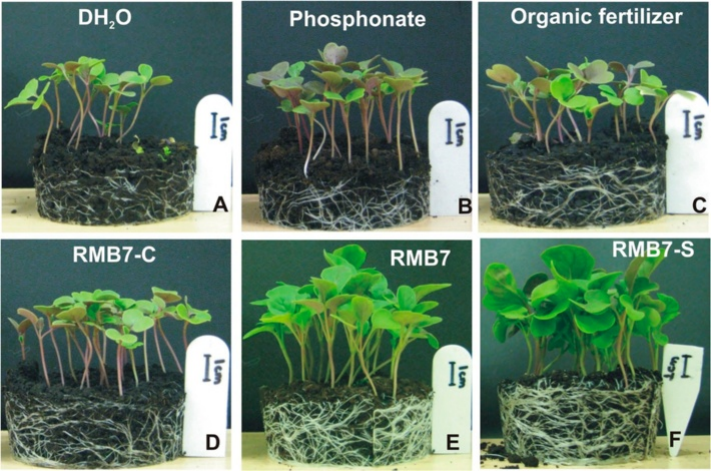
**Representation of plant health and roots extension under the influence of different treatments: (A) Autoclaved distilled water (DH**_**2**_ **O); (B) Fungicide (P); (C) Organic fertilizer (OF); (D) RMB7 cells (C); (E) RMB7 cells + supernatant (CS); (F) RMB7 supernatant (S).**

**Figure 4:**
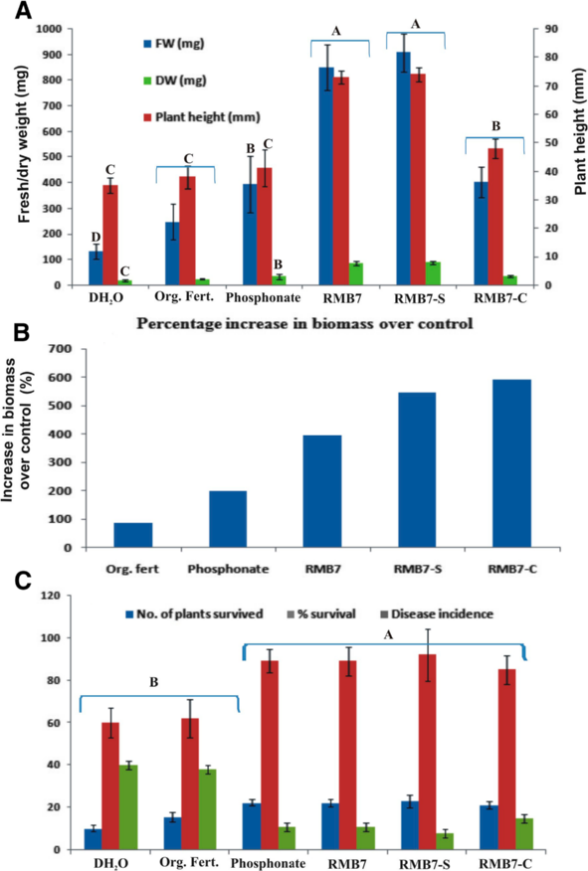
**Response of inoculated Arugula plants in diseased muck soil.** Plant growth **(A)** Biomass **(B)** and survival **(C)** after treatment with autoclaved distilled water (DH_2_O); Fungicide (P); Organic fertilizer (OF); RMB7 cells + supernatant (RMB7); RMB7 supernatant (RMB7-S); RMB7 cells (RMB7-C). Different upper case letters indicate significant differences between means of treatments. Means were compared at α = 0.05 (LSD).

Visual observations of the arugula plants after 2 weeks of inoculations (Figures 2 and 3) showed a significant response of different treatments on plant survival as well as shoot and root biomass production. The plants inoculated with RMB7 cells along-with supernatant (CS) or supernatant alone (RMB7-S), showed improved growth with dense and large green leaves (Figures 2 and 3), heavily proliferated root system (Figure 4), significantly higher plant height and biomass (Figure 4A), as compared to control arugula plants, plants treated with organic fertilizer (OF), pesticide (P), the alone cells of RMB7 (RMB7-C) without supernatant. Increase in plant biomass over control was 88% with organic fertilizer, 199% with pesticide, 397% with inoculation of RMB7 cells alone, 547% with RMB7 cells along with supernatant CS and 592% with RMB7 supernatant alone (Figure 4B).

Arugula plants grown in uninoculated (control) muck soil showed 61% survival while inoculation of arugula either with RMB7 cells along with supernatant (CS), supernatant alone (RMB7-S) or cells alone (RMB7-C) increased plant survival above 85% (Figure 4C). Although the maximum plants (92%) survived in RMB7-S treated pots was slightly higher than the pesticide treated plants (89%) but the growth rate was significantly higher in previous case. The plants treated with organic fertilizer (OF) exhibited no improvement in plant survival over control (Figures 2, 3 and 4A, B, C).

### T-RFLP-analysis of rhizosphere communities under natural pathogen pressure

**Figure 5:**
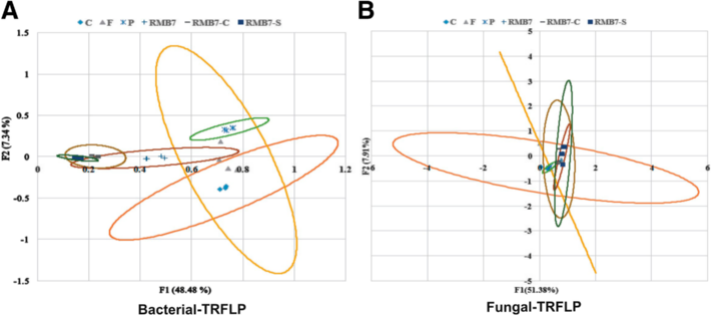
**Principal Component Analysis for PCR-amplified T-RFLP data showing a three-dimensional separation of Arugula rhizosphere bacterial (A) and fungal (B) communities according to overall similarity after inoculation with different treatments of RMB7.** Clustering (indicated by different ellipses) denote significance (p <0.001) for clustering whereas separation indicates low similarity. Axis 1 represents 48.48% **(A)** and 51.38% **(B)** of the variance while Axis 2 represents 7.34% **(A)** and 7.91% **(B)** of the variance.

TRFLP analysis of samples collected from rhizosphere of different treatments was done, with the aim, to study the variation in microbial and fungal community. TRFLP dataset was analyzed by using Principal Component Analysis (PCA) to view transformed microbial community assemblages in a two dimensional space. The PCA plot of bacterial TRFLP showed that microbial communities of RMB7 treatments (RMB7, RMB7-C and RMB7-S) were grouped together (Figure 5: graph A), whereas treatments C, F and P communities were grouped relatively separate from RMB7 treatments which suggested that RMB7 treatments changed the rhizosphere communities as compared to the other three treatments, which might be due to the establishment of RMB7 in the rhizosphere and vicinity. Ultimately, the results of TRFLP analysis suggested that RMB7 treatments were major drivers of community shift and disease control in plant inoculation studies. The results of Fungal PCA plot showed that fungal communities of all the treatments were grouped together (Figure 5: graph B).

### Mass spectrometric analysis of lipopeptides

**Figure 6:**
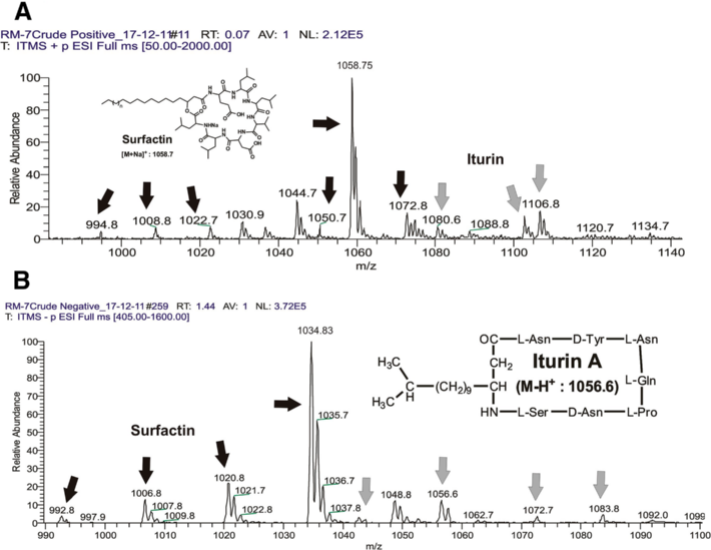
**LCMS chromatograms representing surfactin and iturin molecular ion species; Positive ion mode analysis (A) and Negative ion mode analysis (B).**

Lipopeptides produced by the RMB7 strain were first analyzed through ESI-MS direct injection in positive full scan mode (Figure 6A). The first set of peaks observed were belonged to surfactin family with strong signals at *m/z* 1030.8, 1044.8 and 1058.6 corresponding to sodiated surfactin homologues ions [M + Na]^+^ and weak signals of [M + H]^+^ of these surfactin variants corresponded at *m/z* 994.8, 1008.8, 1022.8 and 1050.6. Negative full scan mode spectra revealed four peaks at *m/*z 992.9, 1006.8, 1020.9, 1034.8 and 1048.8 corresponding to the deprotonated molecules [M-H]^−^ (Figure 5B). These molecules represented the homologues with acyl chains C12, C13, C14, C-15 and C16 respectively (Figure 6 and Table 1).

**Table 1:** **Lipopeptides produced by** ***Bacillus*** **sp. strain RMB7 as detected by ESI-MS**

**Metabolites**	**Exact Mass** ***m/z***	**Observed Peaks** ***m/z***			**Homologue**
		**[M + H]** ^**+**^	**[M + Na]** ^**+**^	**[M-H]** ^**−**^	
**Surfactin**	993	994.8	1008.7	992.9	C-12
	1007	1008.8	1030.8	1006.7	C-13
	1021	1022.8	1044.8	1020.9	C-14
	1035	1036.8	1058.8	1034.8	C-15
	1049	1050.6	1072.6	1048.7	C-16
	1063	-	1086.8	1062.7	C-17
**Iturin A**	1043	-	1066.7	1042.7	C-14
	1057	-	1080.8	1056.7	C-15
	1073	1074.8	1096.7	1072.6	C-16
	1084	-	1106.7	1083.7	C-17

**Figure 7:**
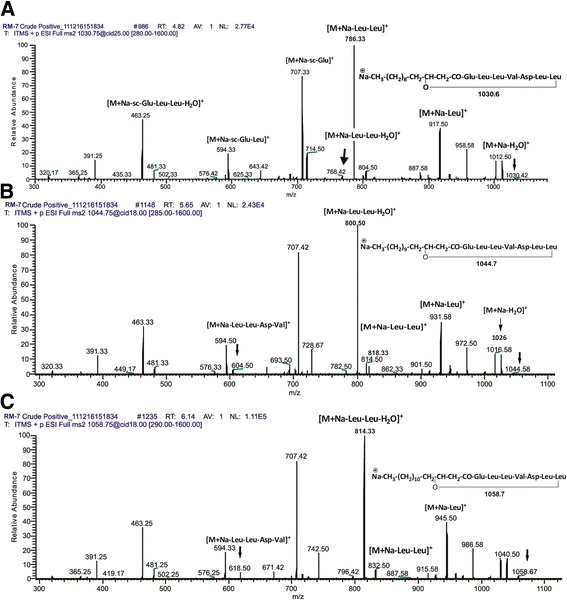
**Fragmentation pattern and product ion spectra of sodiated molecules of surfactin at** ***m/z*** **: (A) 1033.83; (B) 1044.83; (C) 1058.75.**

The sodiated molecules at *m/z* 1030.8, 1044.8 and 1058.6 were subjected to tandem mass spectrometric (MS/MS) analysis due to their higher ion abundance. Fragmentation pattern showed two series of product ions, first series (*m/z* 714 to 1012) contained fatty acid chain and N- terminal ions which is due to loss of amino acids, while the second series (*m/z* 320 to 707) contained the peptidic moiety (without fatty acid chain) inside the C-terminal product ions (Figure 7A). The product ions of molecule *m/z* 1030.8 were observed as: *m/z*320.17, 391.25, 463.25, 481.33, 594.33, 707.42, 786.33, 917.50, 958.58, 1012.50 (Figure 7A).

The product ions of molecules *m/z* 1040.8 and 1058.6 showed exactly the similar pattern of daughter ions from *m/z* 320–707 as that of m/z 1030.8, however, the daughter ions ranged from m/z 714 to upwards varied with *m/*z of 14 but followed the consistent variation pattern, confirming the difference of a methylene group (−CH_2_) variation in the fatty acid side chains among these lipopeptides of surfactin family (Figure 7 A, B & C).

**Figure 8:**
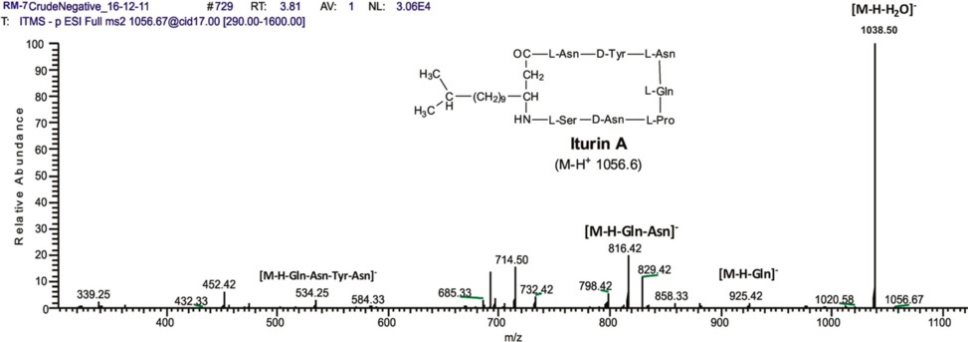
**Fragmentation pattern of molecule of iturin A at** ***m/z*** **[M]- 1056.67.**

**Figure 9:**
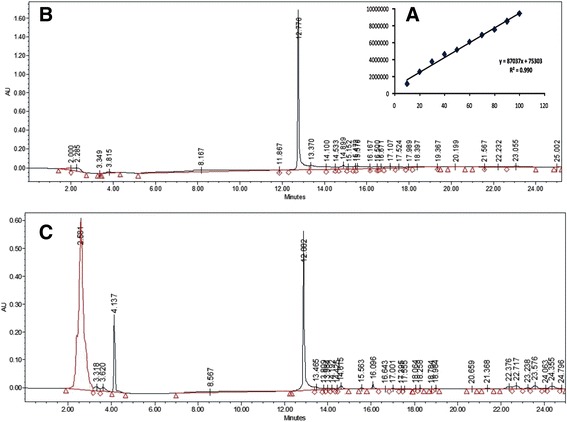
**Standard curve (A), HPLC chromatogram of Iturin A (B) and culture supernatant of** ***Bacillus*** **sp. strain RMB7 (C) showing the production of iturin A.**

The peaks of iturin were apparent in full scan mode (Figure 6) at both positive and negative ionization modes. ESI-MS analysis revealed four main signals of [M-H]- at *m/z* 1042.6, 1056.6, 1072.7 and 1083.8. The molecular weights of these molecules were in agreement with molecular weights of iturin A containing an acyl chain with C14, C15, C16 and C17, respectively (Figure 6B and Table 1). The sodiated ions of iturin A *m/z* 1066, 1080.6 and 1106.8 were evident at positive mode which corresponded to C14, C15 and C17 fatty acyl chains present in iturin A variants molecules, respectively (Figure 6A). Furthermore, the iturin A presence was confirmed by the tandem mass spectrometry of m/z 1056.6, which gave the confirmatory daughter ions peaks (Figure 8). HPLC analysis showed that RMB7 was able to produce 25 μg/mL Iturin A using the described conditions (Figure 9).

### Amplification of genes for lipopeptides

**Figure 10:**
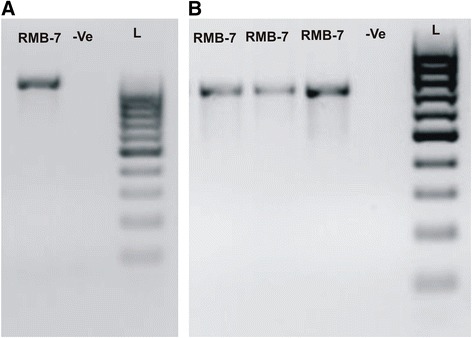
**Amplification of (A)** ***ituD*** **and (B)** ***sfp*** **genes, of** ***Bacillus*** **sp. strain RMB7.-ve represents blank and L represents 1 KB ladder.**

The *sfp* gene has been reported as marker for identification of surfactin producing *B. subtilis* [24]*.* PCR amplification of *sfp* gene fragment (675 bp) and *ituD* gene fragment (1,203 bp) in RMB7 showed positive results (Figure 10). The *ituD* gene encodes a putative malonyl coenzyme A transacylase, which results in production of antibiotic iturin A. These results confirmed that *B. subtilis* RMB7 harbours the genes required for surfactin and iturin A biosynthesis.

## Discussion

*Bacillus* spp. are omnipresent in nature, functionally versatile and generally considered as environmentally safe microorganisms, having a range of agricultural, industrial and environmental applications [25-30]. The bacterium has immense potential for production of antibiotics useful for plant protection and substances important for plant growth [30]. The cyclic lipopeptides are considered to be the most important compounds produced by *Bacillus* that make them such a versatile tool for use in plant disease control [1].

This study describes the isolation of *Bacillus* sp. strain from geographically remote region of foot hills of Himalayan range (maize fields of Rawalakot, Azad Jammu & Kashmir). The isolate termed RMB7, showing best antagonistic activity, was subjected to detailed polyphasic characterization. This strain was confirmed as *Bacillus* sp. through 16SRNA technique but 16S rRNA sequence showed 97% homology to three different subspecies so additional phylogenetic markers or DNA-DNA hybridization can resolve exact specie status. This bacterium exhibited high activities as a biocontrol agent against various fungal phytopathogens and plant growth promoting traits *in vitro.* Strain RMB7 showed >70% inhibition of mycelia growth of the fungal pathogens tested *in vitro* (Figure 1) of which maximum antagonism *i.e.*, 85% was exhibited against *P. irregulare.* The previous *in vitro* studies have shown that *B. subtilis* (strain CU12) can inhibit mycelial growth of *Alternaria solani* (53%), *Botrytis cinerea* (25%), *Fusarium sambucinum* (39%), *Pythium sulcatum* (20%). On contrary to our studies, the main antifungal agent was identified as cyclic dimer of 3-hydroxypropionaldehyde. The PGPR activities of this strain were not explored [36]. Similarly, another *B. subtilis* (named Y-1) was evaluated as potential biocontrol agent against various fungal strains from *Fusarium* spp*.* It exhibited maximum antifungal activity against *Fusarium oxysporum* (64.5%). The antifungal active metabolites were not analyzed [37]. The high *in vitro* antifungal activity shown by RMB7 (Figure 1) in this study may be attributed to the collective effect of multiple antifungal metabolites including diffusible antibiotics, siderophores and lytic enzymes including protease, chitinase, cellulase produced by this strain or simply by the higher production of antifungal lipopeptides.

*In planta*, biocontrol experiment was conducted in field soil, which was unable to produce marketable carrots due to high disease incidence. RMB7 showed the significant biocontrol activity under natural fungal pathogen pressure (Figures 2, 3 and 4). Isolations made from infected carrots harvested from this field provided a *Pythium irregulare* which was identified by sequencing (unpublished data: Ali and Lazarovits, 2013). *P. irregulare* is highly pathogenic to wide range of cereal and legume hosts, causing significant losses in yield as well as vigour worldwide [38]. It is found in broad eco-geographic range and exhibits considerable pathogenic variations. The pathogen is adapted to diverse cropping patterns in cold, humid and temperate climates and is highly persistent in nature. It is the main cause of pre-emergent blight and post-emergent stunting of crops globally [39].

A *Pythium-*susceptible variety of Arugula (*Eruca vesicaria* var. Roquette) was used as model plant. Arugula is grown as mini-green and is often used as salad after two weeks of growth. Growth room data showed the ability of RMB7 (both culture as well as supernatant) to control the disease and improved the survival of Arugula plants in a soil, naturally infested with *Pythium*. RMB7-CS, RMB7-S and RMB7-C treated plants showed similar survival rate as that of P-treated plants but plant biomass was far less in case of later (Figure 4A, B). OF-treated plants although survived under pathogen pressure, but survival rate were significantly low and plants were weak and shorter as compared to RMB7 treatments (Figures 2 and 3). This shows that RMB7 not only supported to control disease but also facilitated plant growth as well (Figures 2, 3 and 4). Studies conducted by Weller et al., (1997) have shown that the strain (L324-92) belonged to *Bacillus* sp*.,* exhibited *in vitro* and *in vivo* antagonistic activities against different fungal strains including *P. irregulare* and *P. ultimatum*, when tested in wheat plants, but the level of antifungal activity or percent inhibition was not reported [40]*.* A recent study have shown 27%-50% suppression of *Pythium* damping-off and root rot of cucumber seedlings along-with 113%-184% increase in plant fresh weight by combined application of two *Pseudomonas* spp. and *B. subtilis* [41]. But the inoculation of *B. subtilis* RMB7 alone resulted in 400–600 folds increase in plant biomass and >85% disease suppression in Arugula plants showing extensively high potential for disease control as well as plant growth promotion. Disease control activity of RMB7 (cells only) may be attributed to the higher production of antifungal metabolites and the collective action of various factors *i.e*., chitinase, cellulase, siderophores that have a profound role in disease suppression along with cyclic lipopeptides [42,43]. Although, *B. Subtilis* antagonistic activity have been reported on different plants but this study is the first report showing higher level of *in vivo* antagonistic activity of *B. Subtilis* against *P. irregulare* on Arugula plants.

The extensively proliferated root system developed due to the inoculation of RMB7 (Figure 4) may be attributed to its ability to produce IAA which has a classical role in enhancing plant root growth [44]. IAA-production along-with P-solubilization facilitates plant’s nutrient acquisition while ACC-deaminase activity decreases the plant ethylene levels and increase plant’s tolerance to biotic and abiotic stresses [45]. The tested strain RMB7 has shown IAA production (8 mg. L^−1^), P-solubilization (50.6 mg. L^−1^ tri-calcium phosphate) and nitrogen fixation activities (reduced 601ηmol acetylene h^−1^/vial). All these factors may have contributed in improving the growth rate of Arugula plants inoculated with RMB7.

TRFLP data taken after two weeks of RMB7 inoculation revealed that bacterial communities in different treatments tend to converge along a few dominant species (Figure 5). PCA showed significantly different bacterial community profile of each inoculated treatment most of which are specific to that treatment. These findings supported the fact that disease control or the plant growth promotion exhibited by RMB7-treated plants (supernatant, cells or both) is exclusively the result of the inoculated bacteria which have disturbed the original ecological balance of soil microbial community temporarily. Further studies however, are necessary to validate the fact whether the RMB7-induced ecological shift is transient or stable.

Studies have shown that antibiosis is the underlying mechanisms of biocontrol in most of the *Bacillus* spp. against many plant and fruit diseases caused by soil born and aerial fungi [46]. The antagonistic activity exhibited by RMB7 supernatant showed that antibiosis might be the mechanism for disease control. The detailed analysis of RMB7-supernatant through ESI-MS technique showed the presence of surfactin and iturin A compounds (Figure 6), which are major bioactive metabolites produced by *B. Subtilis* [16,17]. Surfactins and iturins are the cyclic lipopeptides composed of α-amino acids linked to β-hydroxy fatty acids and have potential biotechnology and biopharmaceutical applications [11,12].

The RMB7 strain was able to produce various homologues of surfactins and iturins A compounds, which were well ionized at positive ESI mode, producing the peaks ranged from *m/z* 994.8-1106.8 (Figure 6A) correlated to protonated and sodiated ion species. At negative ESI mode most of these species were also identified (Figure 6B). These homologues of surfactins and iturins A were further confirmed through tandem mass spectrometry (Figures 7 and 8). The surfactins homologues only vary at the methylene (−CH_2_-) chain length of *β*-hydroxy fatty acyl group and ranged from C_12_-C_17_ (Table 1). While the iturin A homologues demonstrated the variations in methylene chain length ranged from C_14_-C_17_. These ESI-MS/MS structural data agreed with the previously mass spectrometric published data of these lipopeptides [47]. HPLC analysis revealed that RMB7 culture can produce iturin A upto 25 μg. mL^−1^ in the culture medium (Figure 9).

PCR analysis of *B. subtilis* strain RMB7 exhibited presence of gene clusters directly involved in the synthesis of the cyclic lipopeptides surfactin and iturin (Figure 10). Genes for lipopeptides are common to multiple biocontrol strains that have been commercialized and genomes with such genes have demonstrated enhanced capacity to produce antibiotics that inhibit the growth of fungal root pathogens [24,48].

Both surfactin and iturin are well-known for their strong antifungal effect against different phytopathogenic fungi [19]. Surfactins are reported to positively influence cell spreading, swarming and biofilm formation and thus may globally favour plant root colonisation of bacteria. Iturin A on the other hand is strongly haemolytic and displays strong *in vitro* and *in vivo* antifungal action against wide range of fungal phytopathogens [19,20]. The evidence of these two bioactive compounds in RMB7 makes it a useful organism in field of biocontrol and proved it a potent strain when tested under natural pathogen pressure *in planta*.

## Conclusions

The bacterial strain termed RMB7 was isolated from maize fields of geographically remote area located in the foothills of Himalayan range. This bacterium was identified as the *B. subtilis* through 16S *rRNA* sequence analysis. This strain induces the plant growth promoting activities such as the production of growth promoting hormones (IAA), P-solubilising as well as nitrogenase activities. Moreover, it exhibited profound antifungal activities against a variety of fungal pathogens causing different diseases to various crops. This bacterium showed the production of cellulase, chitinase, siderophores, antibiotics *in vitro* and demonstrated strong biocontrol activity against *Pythium irregulare* infested arugula plants *in vivo*. It produces two important families of cyclic lipopeptides that are of prime interest in the plant-microbe beneficial relationship as well as have plant-growth promoting potential. All these physiological, biochemical, and genetic analyses collectively supported the importance of RMB7 as user-friendly biofertilizer and biopesticide agent. This strain has exhibited multifaceted beneficial characteristics and hence can be an ideal plant growth promoting as well as biocontrol agent, for its integrated use in disease and nutrient management strategies for improvement of various crops cultivated in South Asian region. This bacterium may be further explored for its applications in different crops at global level.

## Materials and methods

### Fungal species and culture conditions

*Fusarium oxysporum* and *Fusarium moniliforme* were obtained from First Fungal Culture Bank (Lahore, Pakistan). *Colletotrichum falcatum* was provided by Shakarganj Sugar Research Institute, (Jhang, Pakistan). *Rhizoctonia solani, Aspergillus niger, Aspergillus flavus* and *Colletotrichum gloeosporioides* were obtained from the culture collection of the Microbial Physiology Lab, NIBGE*. Pythium irregulare, Pythium ultimatum* and *Alternaria alternate* were taken from fungal culture collection of A & L Biologicals laboratories, London, ON, Canada. Fungal cultures were maintained on Potato dextrose agar (PDA) at 30 ± 2°C.

### Isolation of antagonistic bacteria

The bacterial strain RMB7 was isolated from the rhizosphere soil (1 g) of healthy maize plants growing in Rawalakot (33°51'32.18"N, 73° 45'34.93"E; elevation 1638 m; annual rain fall 1300 mm; humid subtropical), Pakistan using serial dilution plating [49] on Luria-Bertani (LB) agar plates. The isolate was identified through PCR analysis of 16S rRNA gene as described in previous study [50] using conditions as mentioned in Table 2. Culture was maintained on LB Agar at 28 ± 2°C, inoculums was prepared in LB-broth.

**Table 2:** **Primers and PCR conditions used for TRFLP and amplification of surfactin and iturin from** ***Bacillus*** **sp. strain RMB7**

**Gene/Antibiotic**	**Primer**	**Primer sequence (5’-3’)**	**PCR profile (30 cycles each)**	**Reference**
**16S rRNA**	fD1rD1	AGAGTTTGATCCTGGCTCAG	Denaturing: 95°C 2 min.	[51]
			Annealing: 55°C 30 sec.	
		AAGGAGGTGATCCAGCC		
			Extension: 72°C 5 min.	
**Surfactin**	Sfp-f	ATG AAG ATTTACGGAATTTA	Denaturing: 94°C 1 min.	[52]
			Annealing: 48°C 1 min.	
	Sfp-r	TTATAAAAGCTCTTCGTACG		
			Extension: 72°C 1 min.	
**Iturin A**	ituD-f	ATG AAC ATCTTGCCTTTTTA	Denaturing: 94°C 1 min.	[53]
			Annealing: 50°C 1 min.	
	ituD-r	TTATTTTAAAATCCGCAATT		
			Extension: 72°C 1.5 min.	
**TRFLP-16S rDNA**	VIC-63 F	CAGGCCTAACACATGCAAGTC	Denaturing: 94°C 30 Sec.	[53,54]
			Annealing: 56°C 30 Sec.	
	FAM-1389R	ACGGGCGGTGTGTACAAG		
			Extension: 72°C 2 min.	
**TRFLP-Fungal ITS**	FAM -ITS1	TCCGTAGGTGAACCTTGCGG	Denaturing: 94°C 30 Sec.	[55]
			Annealing: 55°C 30 Sec.	
	VIC- ITS4	TCCTCCGCTTATTGATATGC		
			Extension: 72°C 30 Sec.	

### *In vitro* inhibition of mycelia growth

Dual culture assay described by Sakthivel and Gnanamanickam, [56] was used to determine the antagonistic activity of RMB7 against fungal pathogens. Fungal mycelia plugs (5 mm^2^) were placed near one edge of potato dextrose agar plates and pure colonies of RMB7 were streaked towards the opposite side. The plates inoculated with fungal discs alone and with sterile water instead of bacteria were set as controls. The plates were incubated at 28 ± 2°C for 36 hrs. Results were expressed as percentage inhibition of the growth of fungal mat in the presence and absence of RMB7 as described previously [57].

### Assays for antifungal metabolites

To assess the production of diffusible antibiotics, 20 μL bacterial suspensions (1 × 10^7^ cfu mL^−1^) was inoculated in the centre of PDA plates which were covered with sterile cellophane membrane. After incubation for 36 h at 28 ± 2°C, the membrane with the grown RMB7 was removed and the plate was inoculated in the middle with a 5-mm^2^ disk of fungus. Plates were further incubated for 72 h at 30 ± 2°C and observed for the growth of the fungus. Volatile antibiotics were detected by adding bacterial suspension (1% [v/v], 1 × 10^7^ cfu mL^−1^) to petri dishes containing molten PDA. The plates were allowed to solidify and a disk of fungus (5 mm^2^) was placed at the center of another Petri plate containing PDA. The two plates were placed face to face, avoiding physical contact between the pathogen and the bacterial suspensions, sealed tightly with parafilm to prevent loss of volatiles formed and incubated at 28 ± 2°C for 4 days. The growth of the fungus was measured and compared to control plates containing autoclaved distilled water in place of bacterial inoculum. Hydrocyanic acid (HCN) production was assessed by the method of Lorck [58]. Protease and chitinase activities were determined by streaking pure colonies on skim milk agar plates [59] and chitin agar plates [60] respectively. Cellulase activity was examined on carboxymethyl cellulose (CMC)-agar plates [61]. Plates were incubated at 28 ± 2°C for 4–5 days. The formation of a clear zone around the bacterial colonies was considered as a positive result for the respective enzyme activities. Siderophore production was determined using Chrome Azurol S (CAS) assay [62].

### Assay for plant growth promoting potential

Phosphate solubilization was determined and quantified as described previously [63,64]. IAA was detected and quantified through high-performance liquid chromatography as described earlier [50,65]. Nitrogenase activity was determined by acetylene reduction assay (ARA) using a gas chromatograph (Thermoquest, Trace G.C, Model K, Rodono Milan, Italy) using a Porapak Q column and a H_2_ -flame ionization detector (FID)as described [66,67]. The ability to use 1-aminocyclopropane-1-carboxylic acid (ACC) as a sole nitrogen source was assessed in 5 mL DF salt minimal medium [68] containing 3 μL of 0.5 M ACC. Biofilm formation was assessed by micro-titre plate assay as described [69].

### Growth room studies

Biocontrol efficacy of RMB7 was tested in growth room *in planta* using muck soil. The soil was collected from carrot field (Kettleby, ON, Canada,) which could no longer produce a marketable carrot due to carrot root dieback disease. The soil was naturally infested with *Pythium irregulare* and was characterized as bad soil having organic matter: 76.1%; pH: 6.1; CEC: 17.6 meq/100 g; P: 32 ppm; K: 71 ppm; N: 29 ppm, Mg: 315 ppm. Seeds of a *Pythium-*susceptible variety of Arugula (*Eruca vesicaria* var. Roquette) were obtained from seeds collection facility at A & L Biologicals Laboratories, Canada. Germination of the seeds and plant growth rates were determined in autoclaved sand as 100% viable. Inoculum of RMB7 was prepared by transferring a pure single colony into LB broth. The bacteria were grown for 36 h in an incubator shaker (100 rpm) at 28 ± 2°C. Cells were pelleted by centrifugation at 8000 rpm at 4°C for 5 minutes, re-suspended in equal volume of saline and diluted to 1 × 10^7^ CFU mL^−1^. The supernatant was filtered (0.45 μm syringe filter) and used as inoculum. The water holding capacity (% WHC = 80) of soil was measured to adjust the volume of inoculum. Almost 250 g of soil was poured in plastic bags and 50 mL of inoculum was added; mixed thoroughly and placed in the dark for two days at room temperature to allow the bacteria to establish. After two days, 30 g of soil was poured to each paper cup (10 ounce), 20 seeds of arugula were sprinkled on the soil surface and seeds were covered with 20 g of more soil. The cups were covered with lids and incubated in a growth room with 25% humidity, 16 hour photoperiod (200 μM m^−2^ sec^−1^ at pot height with incandescent and fluorescent lights), with 25°C day and 22°C night temperature. The covers were removed when seedlings emerged after 2–3 days. Six different inoculations of soil were carried out including: RMB7 cell suspension (C), filtered supernatant (S), RMB7 culture (Cells + supernatant) (CS), an organic fertilizer (OF) and fungicide (Calirus 150 known to inhibit *Pythium*, contained 12% phosphonate) (P). The recommended dose of the organic fertilizer and the fungicide used in this study was 1:200 (v/v) pure products: double distilled water, respectively. Soil treated with autoclave distilled water was used as the untreated control. The experiment was set up in completely randomized design (CRD) with four replicates per treatment. The experiment was repeated twice for comparing results. Plants were watered daily, harvested after 14 days and plant height, fresh weight, dry weight was measured and percentage survival, total biomass were calculated. The data of 25 plants from each replicate was averaged to get mean value. On the whole 100 plants were sampled for each treatment. The effect of inoculation was determined by analysis of variance (ANOVA) using STATISTIX 8.1 software. Means were compared by applying Tukey test at α 0.05.

### TRFLP-analysis of RMB7-inoculated rhizosphere communities under natural pathogen pressure

From the rhizosphere of different treatments of plants inoculated with RMB7 under natural pathogen pressure, tightly attached root soil was collected. Metagenomic DNA from the 200 mg of each soil sample was extracted using Norgen Genomic DNA Isolation kit (Norgen Biotek Corp., Thorold, Ontario, Canada) following the manufacturer’s protocol. The bacterial community profiles were investigated by analysing partial 16S-rDNA gene sequences while fungal community profiles were investigated by analyzing ITS gene sequences using labelled primers (Table 2). For both TRFLP analyses, 50 μl of PCR mixture was prepared in dH_2_O using 10x PCR buffer (5 μl), 25 mM MgCl_2_ (3 μl) (Invitrogen, Carlsbad, CA, USA), 4 mM dNTPs (2.5 μl), 10 μM primers (1 μl of each) and 5U/ μl Taq DNA polymerase (0.25 μl) (Invitrogen). In 16S *rRNA* PCR mixture, 20 mg/ml BSA (1.25 μl) was also added. The PCR conditions were set up as described (Table 2) along-with 5 min initial denaturation at 94°C and final extension at 72°C for 7–10 min in a Bio-Rad CFX Connect® real-time thermal cycler. The PCR-amplified products were separated on 1% agarose gel, purified using a DNA Clean and Concentrator −5 kit (Zymo Research Corporation, Irvine, CA, USA) and 13 μl of PCR product was digested with 0.5 μl of 5 U of *HhaI* (New England Biolabs) restriction enzyme for 3 h at 37°C. The reaction was deactivated by incubating at 65°C for 5 min.

The fluorescently labelled terminal restriction fragments (TRFs) were separated by capillary electrophoresis in an automated DNA sequencer equipped with fluorescence detector (Applied Biosystems 3730 DNA Analyzer) at the University of Guelph. The length (in base pairs) of the TRFs were determined by comparing with internal standard (1200 bp) using GeneMarker® software V2.4.0, with a cut off size of 39 bp and peak intensity detection range was 40–30,000. For each inoculation treatment, three independent plant DNA-metagenomes were analyzed and microbial community structures were estimated by counting the number of peaks generated from each metagenomic DNA sample, and averaged by plant growth stage and crop field. The abundance of particular TRF was determined by measuring the fluorescence unit. Binary data were exported to XLSTAT 2013 software (AddinSoft, France) and principle component analysis (PCA) was performed [70] to determine microbial community profiles.

### Extraction and purification of lipopeptides

To identify the lipopeptides, RMB7 was inoculated to 100 ml LB broth in 250 mL Erlenmeyer glass flask and incubated in incubator shaker at 28 ± 2°C. After 36 h 100 mL of bacterial culture was pelleted by centrifugation (8000 rpm at 4°C for 10 minutes). The supernatant was collected and lipopetides were precipitated after adjusted the pH of medium to 2.0 with 6 N HCl and kept at 4°C for 2 h. The precipitates were recovered by centrifugation (8000 rpm at 4°C for 20 minutes) and dissolved in 2:1 (v/v) methanol: water. The extract was purified by filtering through 0.45-μm syringe filter, dried under vacuum, re-suspended in 2 mL methanol and stored at −20°C.

### Mass spectrometeric analysis

All mass spectrometry analyses were done on LTQ XL Linear Ion Trap Mass Spectrometer (Thermo Scientific, USA) equipped with an ESI source. Samples were injected with a syringe pump at (5 μL/min flow rate). Source voltage and capillary voltage were 4.80 kV and 23 V, respectively, in positive ion mode. Capillary temperature and sheath gas flow (N_2_) were 350°C and 30 arbitrary units, in both scan modes. Data was acquired at both positive and negative total ion full scan mode (mass scan range: *m*/*z* 50–2000). The ESI-mass spectra obtained were used to characterize the surfactants ionization behaviour and [M + H]^+^, [M - H]^−^ and [M + Na]^+^ ions were monitored for iturin A and surfactin. In addition, the ESI-MS/MS fragmentation behaviour of identified peaks was investigated to confirm the structure of Iturin A and Surfactins.

### Quantification of Iturin A by liquid chromatography

The amount of iturin A in the extract was measured by high performance liquid chromatography system (2487 HPLC, Waters, USA) consisted of LCD 600 pump controller, dual absorbance detector and an on-line degasser. Column used in this analysis was YMC-Pack Pro C18 column (250 mm × 4.6 mm, 5 μm particle sizes, YMC, America). Mobile phase A was water (0.1% acetic acid) and mobile phase B was 60% acetonitrile (0.1% acetic acid). Mobile phase A was run for 5 min followed by 25 minutes flow of mobile phase B. The flow rate and the temperature of the column was optimized by using standard Iturin A >90% (Sigma Aldrich, Canada) to allow for appropriate separation of iturin A from any impurities. Standard curve was made by running different concentrations of standard iturin A (10, 20, 30, 40, 50, 60, 70, 80, 90 and 100 ppm). The optimized flow rate was 1.0 mL/min and temperature of column compartment was set at 25°C with injection volume of 10 μL. All the peaks were detected at 230 nm wavelength of UV. Samples were analyzed in three replicates and process was repeated.

### Detection of genes responsible for production of lipopeptides

The total genomic DNA of strain RMB7 was extracted by using CTAB method [71] with some modifications. Primers used for amplification of genes involved in synthesis of surfactin and iturin lipopeptides are shown in Table 2. For each sample 50 μL reaction mixture was prepared containing 2 μL of template, 1 μL of 10 mM each primer, 5 μL of 2 mM dNTP (Invitrogen), 5 μL 10 × PCR buffer, 1.5 μL of 25 mM MgCl_2_ (Fermentas), 0.5 μL of 0.1% DMSO and 0.5 μL of 50U mL^−1^ Taq DNA polymerase (Fermentas). The amplifications were performed using a thermocycler (Eppendorf) with the cycle conditions described in Table 2.
